# Anlotinib plus PD-1 inhibitor in advanced soft tissue sarcoma: a real-world study on treatment patterns and treatment-emergent hypothyroidism

**DOI:** 10.3389/fimmu.2026.1949174

**Published:** 2026-09-11

**Authors:** Fang Wu, Wen-Ting Zhu, Yi-Ze Li, Yi Zheng, Hong-Mei Zhang, Xiao-Wen Wang

**Affiliations:** Department of Clinical Oncology, Xijing Hospital, Fourth Military Medical University, Xi’an, China

**Keywords:** anlotinib, hypothyroidism, immunotherapy, prognostic factors, soft tissue sarcoma

## Abstract

**Background:**

Anlotinib was approved for advanced soft tissue sarcoma (STS) but validated biomarkers and optimal treatment patterns remain unclear. This study evaluated the efficacy, safety and prognostic factors of anlotinib-based regimens, and assessed whether the addition of PD-1 inhibitors is associated with improved outcomes in advanced STS.

**Methods:**

Data from patients with advanced STS receiving anlotinib between January 2019 and December 2024 were retrospectively analyzed. Survival was assessed using Kaplan-Meier and Cox regression. Landmark analyses at 2, 3, and 4 months were performed to address potential immortal time bias. Proportional hazards assumptions were assessed using Schoenfeld residuals, and sensitivity analyses included stratified Cox models and ridge regression.

**Results:**

Of the 227 patients screened, 84 were included in the study. The median follow-up time was 30 months. The median PFS and OS were 10 months and 26 months, respectively. Anlotinib plus immunotherapy regimen achieved the highest ORR (37.5%) and numerically the longest median PFS (20 months) and OS (29 months) among all treatment patterns, though adjusted comparisons versus monotherapy were not statistically significant. Anlotinib maintenance therapy was associated with longer PFS (HR 0.30, 95% CI 0.13-0.66, *P* = 0.003) and OS (HR 0.15, 95% CI 0.05-0.51, *P* = 0.002) compared with monotherapy. Treatment-emergent hypothyroidism was a favorable prognostic factor (PFS HR 0.24, OS HR 0.21, both *P* < 0.001).

**Conclusions:**

The addition of PD-1 inhibitors to anlotinib was associated with numerically longer survival and a high disease control rate in a small subgroup with a higher proportion of immunotherapy-sensitive histologies; these descriptive findings warrant further prospective evaluation. Treatment-emergent hypothyroidism was identified as an independent favorable prognostic factor. Maintenance therapy was associated with prolonged survival, but this finding should be interpreted cautiously due to potential selection bias and violation of the proportional hazards assumption.

## Introduction

Soft tissue sarcoma (STS) is a rare malignancy that originates from the body’s soft tissues, including muscles, tendons, adipose tissue, and blood vessels (1). The incidence reaches 15% of all childhood cancers, but fewer than 1% in adult. The lung is the most common site for advanced STS (2), other potential sites of metastasis include bone, liver and brain. It has been reported that 14.5%-26.5% of STS patients present with metastases at the initial diagnosis, while 40%-50% of patients who underwent localized resection developed distant metastases and showed a 5-year survival of less than 10% (3). Anthracycline-based systemic chemotherapy is recommended as the standard first-line treatment for advanced STS, with response rates of approximately 26%; the median progression-free survival (PFS) was only approximately 6 months and the median overall survival (OS) was 12–16 months (4–6). However, the proportion of chemotherapy-sensitive histological subtypes is low, and chemotherapy toxicities restrict its application. Thus, the exploration of more effective and well-tolerated therapeutic strategies for advanced STS is urgently warranted.

In this context, anti-angiogenic therapy has emerged as another important treatment option. Anlotinib, a multi-kinase inhibitor targeting vascular endothelial growth factor receptor (VEGFR), platelet-derived growth factor receptor (PDGFR), and fibroblast growth factor receptor (FGFR) (7), was approved by the China Food and Drug Administration (CFDA) as second-line therapy in 2019 (8) and subsequently as first-line therapy in combination with chemotherapy in 2025. Beyond its use in chemotherapy combination, anlotinib has also been explored as monotherapy in first-line settings for patients unfit for or refusing chemotherapy, with a phase II trial (9) of 40 patients reporting a median PFS of 6.83 months, an OS of 27.4 months, and a disease control rate (DCR) of 67.5%. Additionally, as maintenance therapy following first-line anthracycline-based chemotherapy, the phase II ALTER-S006 study (n=49) demonstrated a median PFS of 9.1 months (95%CI 5.7-12.5) and a 1-year OS rate of 98.0% (10). These findings collectively establish anlotinib as a versatile backbone for diverse clinical scenarios in advanced STS.

Given the limited durability of anti-angiogenic monotherapy, there is growing interest in combination strategies to augment its efficacy. In this regard, Immune checkpoint inhibitors have demonstrated modest but subtype-restricted activity in STS, with responses predominantly observed in alveolar soft part sarcoma (ASPS) and undifferentiated pleomorphic sarcoma (UPS) (11). Preclinical and clinical evidence suggests that VEGF inhibition may enhance immunotherapy efficacy by normalizing tumor vasculature and reprogramming the immunosuppressive microenvironment, providing a strong rationale for combining PD-1 inhibitors with antiangiogenic agents such as anlotinib (12–14). A single-arm phase II study of anlotinib plus TQB2450 (an anti-PD-L1 inhibitor) in 30 patients with advanced STS reported an objective response rate (ORR) of 36.67%, DCR of 76.67%, and median PFS of 7.85 months, with ASPS subgroup (n=12) achieving an ORR of 75% and median PFS of 23.06 months (15). In light of the above evidence, three distinct treatment strategies emerged for anlotinib in advanced STS: monotherapy, maintenance after chemotherapy, and combination with PD-1 inhibitors. However, the optimal strategy and patient selection criteria, particularly which patients are most likely to benefit from the addition of immunotherapy remain undefined. There are currently no validated predictive indicators to identify which patients may benefit from different treatment patterns as well as its efficacy and safety.

To address this critical clinical issue, we conducted a retrospective real-world study to evaluate the efficacy, safety, and prognostic factors of different anlotinib-based regimens in patients with advanced STS. Specifically, we sought to evaluate the efficacy of PD-1 inhibitors added to anlotinib, and to identify clinically accessible biomarkers that may guide treatment selection and individualize therapy. Although the precise mechanisms linking these factors to immunotherapy response require further investigation, their independent prognostic value in the context of PD-1 containing regimens supports their potential utility for patient stratification.

## Materials and methods

### Data collection

**Figure 1:**
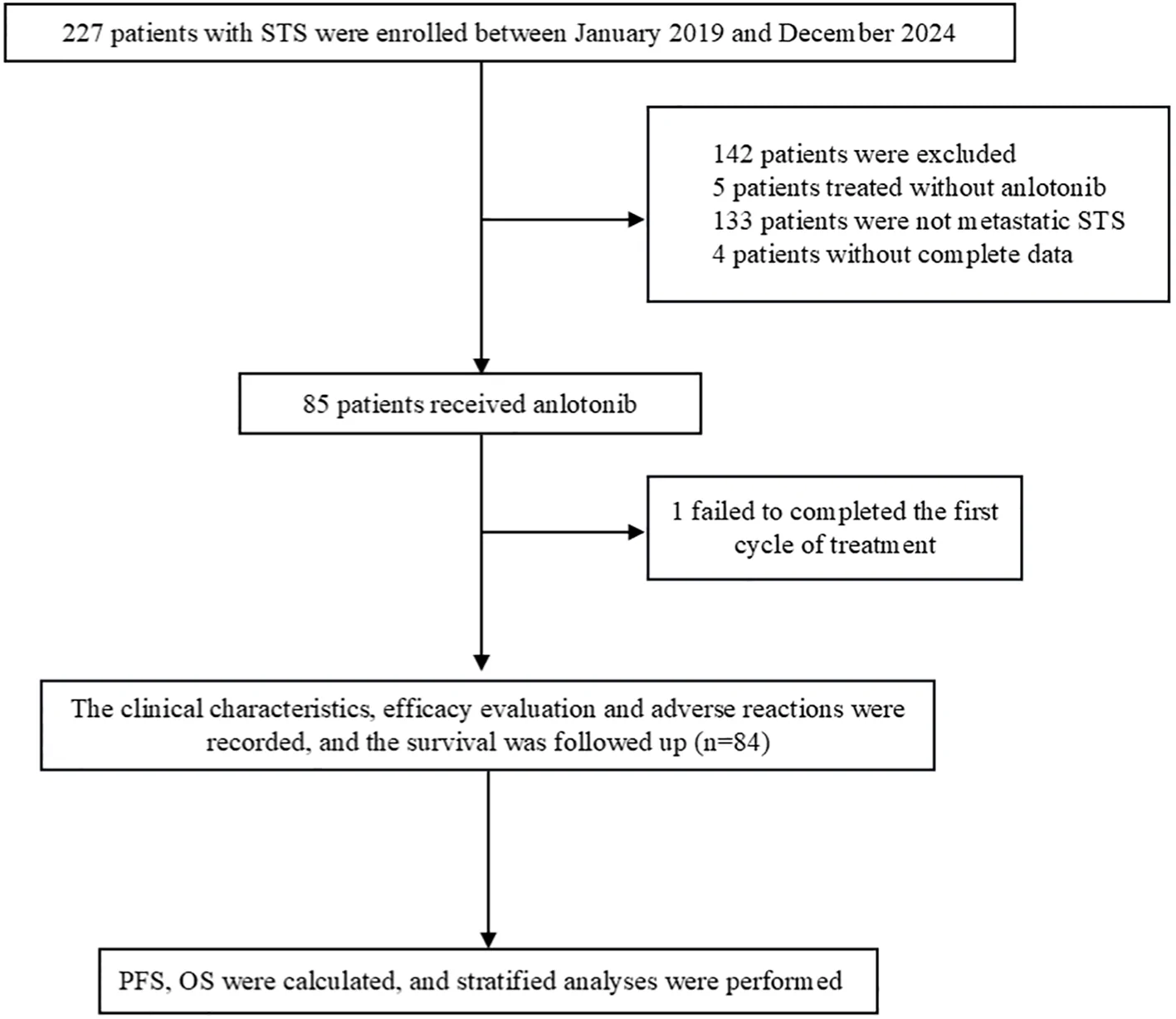
Study profile of patient enrollment and analysis.

We screened the electronic medical records of the STS patients who were hospitalized in the Department of Clinical Oncology at Xijing Hospital of Fourth Military Medical University from January 2019 to December 2024. Clinical data of patients who were diagnosed with advanced or metastatic STS and received at least one cycle of anlotinib treatment were selected. Clinicopathologic baseline data collected for further analysis included gender, age, Eastern Cooperative Oncology Group (ECOG) performance status, pretreatment body mass index (BMI), pathological subtypes, number of metastatic sites, grade according to the FNCLCC (French Federation of Cancer Centers Sarcoma Group) system, primary and metastatic sites, treatment patterns and treatment lines. FNCLCC grade was assigned based on tumor differentiation, mitotic count, and necrosis. For rare subtypes where FNCLCC grading is not routinely applied, the same three parameters were used. The patients were followed up until May 31^st^, 2025, and the PFS and OS were confirmed through hospital records or follow-up calls. Outcome lengths were determined as months from the initiation of anlotinib administration to disease development or death, respectively. At least one observable or measurable lesion according to the Response Evaluation Criteria in Solid Tumors (RECIST) guidelines version 1.1 was required for inclusion. Those cases with no follow-up data after treatment with anlotinib were excluded. The study workflow is outlined in **Figure 1**. The study was approved by the Medical Science Committee of Xijing Hospital of Fourth Military Medical University (Approval Document No. XJLL-KY20212184). Written informed consent was provided by all participants prior to their inclusion in the study. All procedures performed in studies involving human participants were in accordance with the ethical standards of the institutional and/or national research committee and with the 1964 Helsinki declaration and its later amendments. All histological diagnoses were reviewed by dedicated sarcoma pathologists at our institution. For diagnostically challenging cases, molecular confirmation was performed when clinically indicated (e.g., SS18-SSX fusion for synovial sarcoma, ASPSCR1-TFE3 fusion for ASPS, EWSR1 rearrangement for small round cell tumors).

### Treatment methods

Anlotinib was administered orally at 12 mg once daily for 14 days, followed by a 7-day rest period (21-day cycle), with dose reductions to 10 mg or 8 mg permitted for intolerance. Three patients under 18 years of ages (aged 11, 16, and 17 years) received the same adult dosing regimen. PD-1 inhibitors were given intravenously on day 1 of each 21-day cycle. Treatment was discontinued upon disease progression or unacceptable toxicity. In the A+I and chemotherapy combination groups, adverse events were attributed to the most likely causative agent by the treating investigator, based on the known toxicity profiles of anlotinib, PD-1 inhibitors, and the chemotherapeutic agents used, as well as the temporal relationship between drug administration and event onset. Events that were consistent with the known safety profile of anlotinib (e.g., hand-foot skin reaction (HFSR), hypertension, proteinuria, hypothyroidism) were attributed to anlotinib. Events that could not be clearly attributed to a single agent were recorded as combination therapy-related and were not included in the anlotinib-specific adverse event counts.

Anlotinib maintenance therapy was considered for patients who achieved at least stable disease after anthracycline-based chemotherapy, maintained an ECOG performance status of 0-1, and opted for oral maintenance.

Patients were categorized into three treatment groups based on their actual regimen (1): monotherapy (anlotinib alone) (2); maintenance therapy; and (3) combination therapy (anlotinib with other systemic agents). For detailed comparative analysis, combination therapy was further divided into two subgroups: anlotinib-plus-immunotherapy (A+I) and other combination regimens (primarily with chemotherapy).

Chemosensitivity was classified as hypersensitive, moderately sensitive, or insensitive based on the histology-based expectations of relative sensitivity to conventional anthracycline-based chemotherapy, rather than on observed responses in this cohort. This qualitative, ordinal classification was based on published evidence from the British Sarcoma Group (BSG) guidelines (16) and the Chinese Society of Clinical Oncology (CSCO) guidelines for soft tissue sarcoma (17), which group common subtypes according to their anticipated benefit from cytotoxic chemotherapy. Specifically, highly sensitive subtypes were categorized as hypersensitive; moderately high sensitive and moderately sensitive subtypes were categorized as moderately sensitive; and insensitive and extremely insensitive subtypes were categorized as insensitive. This classification was applied to the predominant histological subtype of each tumor; therefore, not all cases of a given histology necessarily fell into the same chemosensitivity category. This classification was supplemented by the patient’s observed response to prior chemotherapy when available, and was used as a clinical framework rather than a formally validated classification system.

Tumor response was assessed by CT or MRI approximately every 6 weeks (every 2 cycles) using RECIST version 1.1. Assessment was performed by the treating oncologist and retrospectively reviewed by a study dedicated radiologist. For patients receiving immunotherapy-containing regimens, if progressive disease was suspected, confirmatory imaging was performed to distinguish true progression from pseudoprogression; however, formal irRECIST assessment was not systematically applied. All patients had measurable disease at baseline and were evaluable for response.

### Outcomes

The primary end point was PFS for anlotinib, secondary endpoints included OS, and safety. PFS was defined as the period from the start of anlotinib monotherapy, maintenance or combination therapy until the date of disease progression. If the subject did not experience disease progression or death, PFS was defined as the period from initiation of treatment to the date of the last confirmed progression-free status. The OS was defined as the period from the start of anlotinib until death due to any cause. Treatment-related adverse events (AEs) were recorded and graded according to the Common Terminology Criteria for Adverse Events (CTCAE) version 5.0.

### Statistical analysis

Age, PFS, OS, and follow-up time are reported as the median and range. Gender, metastatic sites, pathological type, AEs, and tumor response are presented as frequency counts and percentages. The Kaplan-Meier method was employed to represent the survival curves for PFS and OS, and compared by the log-rank test. Variables for the multivariable Cox regression model were selected based on clinical relevance: sex and ECOG performance status as established prognostic factors; treatment pattern as the exposure of interest; hypothyroidism and HFSR as the treatment-emergent factors under evaluation; and BMI for its reported prognostic relevance. The proportional hazards assumption was tested using Schoenfeld residuals, and collinearity was assessed by the variance inflation factor (VIF). A sensitivity analysis was performed by adding a binary histological variable (ASPS/UPS vs. other histologies) to the model. Given the limited number of OS events, penalized Cox regression (ridge) was also applied. Baseline characteristics of patients with and without hypothyroidism were compared using standardized mean differences (SMD). As a further sensitivity analysis, the multivariable Cox model was repeated after excluding patients with unknown hypothyroidism status (n = 9), using the same covariates as the primary model. All data analyses were conducted using SPSS version 28.0 (IBM Corp., Armonk, NY). GraphPad Prism version 9.0.0 and R software (version 4.5.1) were used for plotting survival curves. A two-sided *P*-value of < 0.05 was considered statistically significant. To address potential immortal time bias, landmark analyses were performed at 2, 3, and 4 months. At each landmark time point, patients who had experienced disease progression or death before the landmark were excluded, and the remaining patients were reclassified according to whether hypothyroidism had occurred prior to the landmark. Because the exact date of hypothyroidism onset was not systematically recorded, the timing of hypothyroidism was determined by the first documentation of hypothyroidism in the medical record, used as a proxy for onset. Patients with unknown hypothyroidism status (n=9) were retained as a separate category in all multivariable models. In the landmark analyses, patients were excluded only if they had experienced disease progression or death before the landmark time point. Multivariable Cox regression was then repeated using survival time calculated from each landmark date. For clarity, the hazard ratios presented in **Table 1** are for confirmed hypothyroidism (yes) versus no hypothyroidism (no); patients with unknown status were retained as a separate category in the model but are not displayed.

**Table 1:** Landmark analysis of hypothyroidism.

Landmark	N	PFS HR (95%CI)	*P*	OS HR (95%CI)	*P*
2 months	83	0.21 (0.10-0.45)	<0.001	0.19 (0.08-0.47)	<0.001
3 months	72	0.25 (0.11-0.57)	0.001	0.32 (0.11-0.94)	0.038
4 months	67	0.22 (0.09-0.50)	<0.001	0.30 (0.10-0.91)	0.033

## Results

### Patient clinical characteristics and treatment

We collected 227 patients diagnosed with STS between January 2019 and December 2024 in the Department of Clinical Oncology at Xijing Hospital of Fourth Military Medical University, 142 patients were excluded and 1 patient failed to complete the first cycle of anlotinib. At last, a total of 84 patients with advanced STS who had a history of anlotinib treatment were included in this study. The data cut-off date was May 31^st^, 2025. The median follow-up time for living patients was 30 months (range: 5-71). The median age at baseline was 30 years (range: 11-74), and 40 patients (47.6%) were male, 36 patients (42.9%) had an ECOG score of 0 (no functional impairment), and 48 patients (57.1%) had an ECOG score of ≥1 (with functional impairment). Most of the patients (n=58, 69.0%) were normal weight (BMI 18.5-24.9), 17 patients (20.2%) were overweight (BMI ≥ 25). The trunk, extremities and intra-abdominal were the most frequently observed primary tumor locations (82.1%). 28 patients had lung metastases, 27 patients had extrapulmonary metastasis and 29 patients had multiple metastatic sites (both intrapulmonary and extrapulmonary metastasis). The largest groups of pathological types were leiomyosarcoma (n=14, 16.7%) and UPS (n=12, 14.3%). Based on the FNCLCC grading system, 5 patients (6.0%) were classified as grade 1, 27 patients (32.1%) as grade 2 and 52 patients (61.9%) as grade 3. Seventy-four patients (88.1%) had a history of surgical resection, and 37 patients (44.0%) had experienced radiotherapy.

Regarding the line of therapy, anlotinib was administered as first-line treatment in 43 (51.2%), as second-line in 33 (39.3%), and as third-line or later in 8 (9.5%). Twenty-nine patients (34.5%) received anlotinib monotherapy, 33 patients (39.3%) received anlotinib combination therapy, and 22 patients (26.2%) received anlotinib maintenance therapy after chemotherapy. The 33 cases of anlotinib combination therapy primarily included systemic therapies such as chemotherapy (n=17), immunotherapy (n=16). The chemotherapy regimen applied for STS patients included anthracyclines, ifosfamide, docetaxel, gemcitabine and protein-bound paclitaxel. The immunotherapy regimens included toripalimab (n=9), nivolumab (n=2), and tislelizumab (n=5), all of them were PD-1 inhibitors. The baseline characteristics are presented in **Table 2**. Baseline characteristics stratified by treatment pattern are provided in **Supplementary Table 1**. Imbalances were observed in histological subtype (SMD 1.117), while ECOG performance status, sex, and age were relatively balanced across groups.

**Table 2:** Baseline characteristics of patients.

Characteristics	N (%)	Characteristics	N (%)
Age (years)		Histological subtype	
median	30	synovial sarcoma	8 (9.5%)
Range ≤40	11-74 45 (53.6%)	Fibrosarcoma leiomyosarcoma	10 (11.9%) 14 (16.7%)
>40	39 (46.4%)	UPS	12 (14.3%)
Gender		rhabdomyosarcoma	10 (11.9%)
female	44 (52.4%)	alveolar soft part sarcoma	6 (7.1%)
male	40 (47.6%)	liposarcoma	11 (13.1%)
BMI (kg/m^2^)		angiosarcoma	2 (2.4%)
underweight (<18.5)	9 (10.7%)	epithelioid sarcoma	4 (4.8%)
normal (18.5-24.9) overweight (≥25)	58 (69.0%) 17 (20.2%)	small round cell tumor unclassified sarcoma	4 (4.8%) 3 (3.6%)
ECOG score		Treatment pattern of anlotinib	
0	36 (42.9%)	monotherapy	29 (34.5%)
≥1	48 (57.1%)	combination therapy	33 (39.3%)
Primary site		anlotinib+ immunotherapy	16 (19.0%)
head and neck	8 (9.5%)	other combination therapy	17 (20.2%)
trunk	26 (31.0%)	maintenance therapy	22 (26.2%)
extremities	23 (27.4%)	Treatment lines of anlotinib	
intra-abdominal	20 (23.8%)	1st-line	43 (51.2%)
viscera	7 (8.3%)	2nd-line	33 (39.3%)
Metastatic sites		≥3rd-line	8 (9.5%)
intrapulmonary	28 (33.3%)	Surgery history	
extrapulmonary	27 (32.1%)	yes	74 (88.1%)
multiple sites	29 (34.5%)	no	10 (11.9%)
FNCLCC grade		Radiotherapy history	
1	5 (6.0%)	yes	37 (44.0%)
2	27 (32.1%)	no	47 (56.0%)
3	52 (61.9%)		

### Efficacy and safety

**Figure 2:**
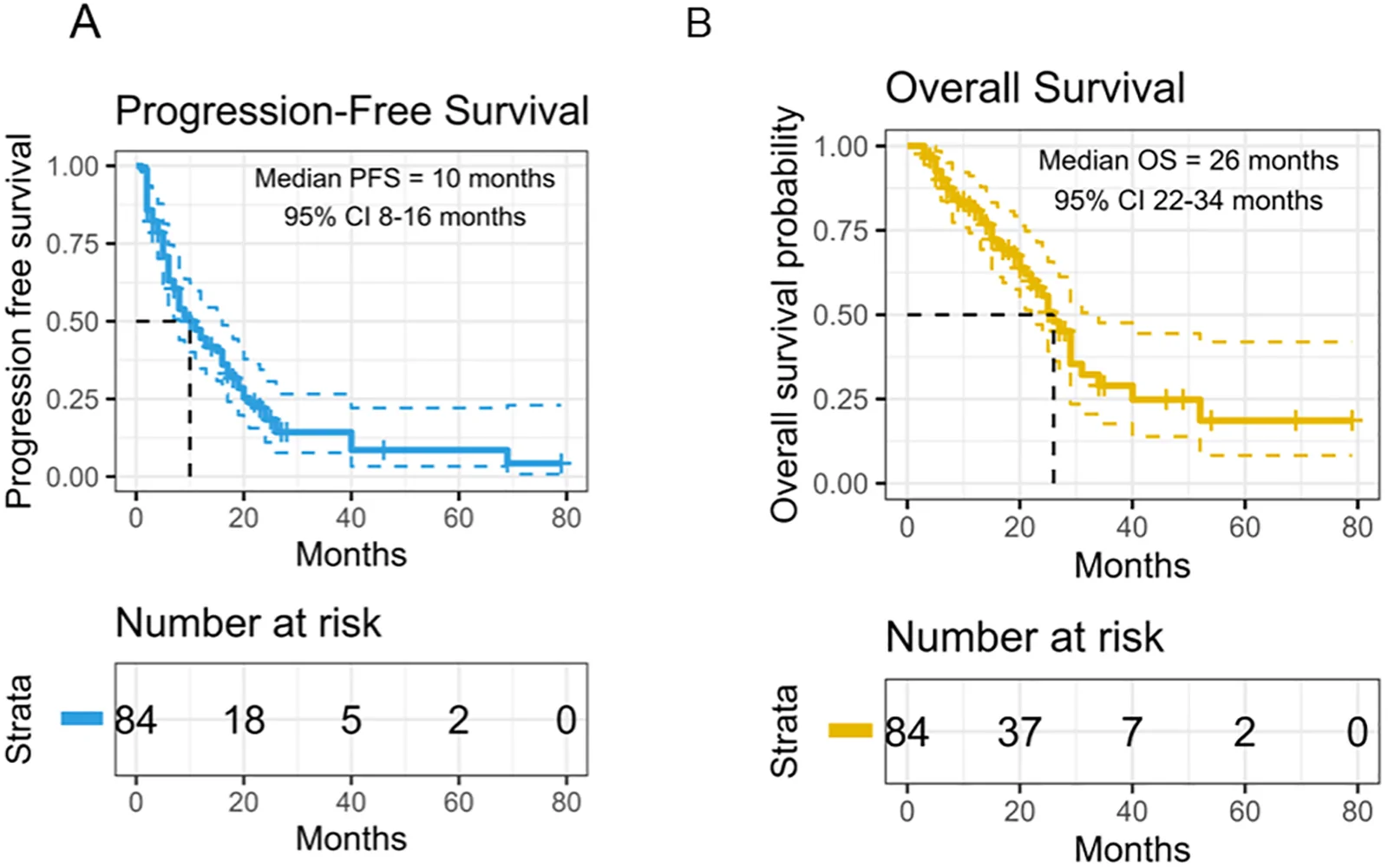
Kaplan-Meier curves of progression-free survival **(A)** and overall survival **(B)** for all patients.

**Figure 3:**
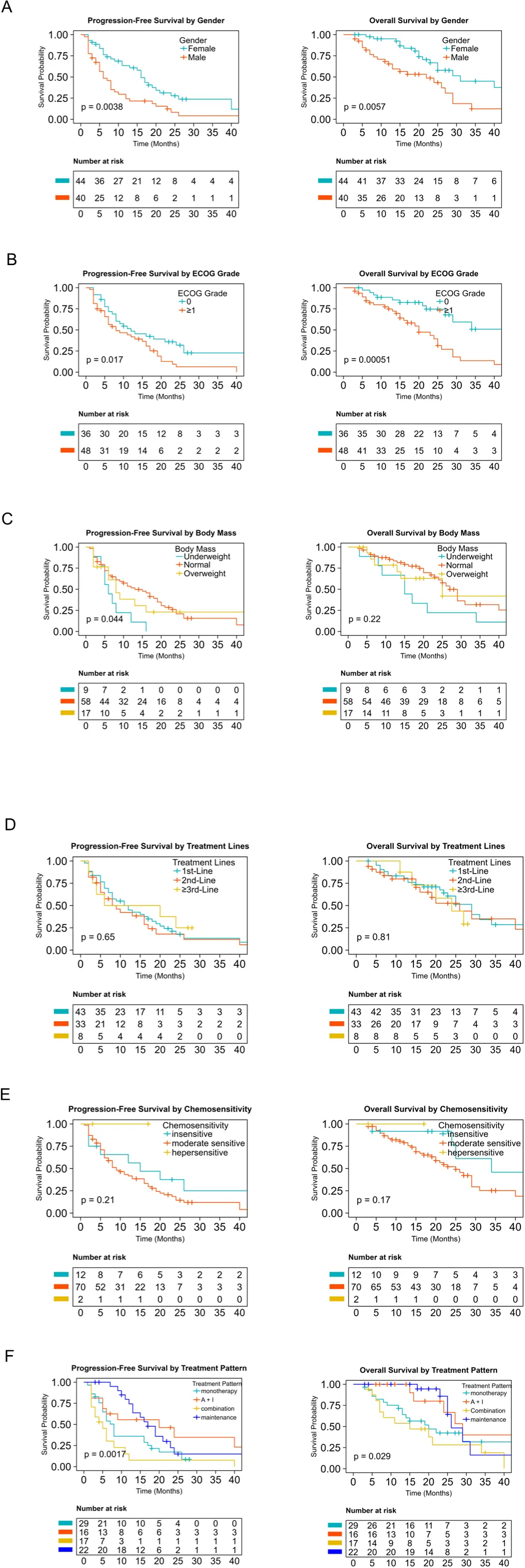
Survival curves of progression-free survival and overall survival according to gender **(A)**, ECOG grade **(B)**, body mass **(C)**, treatment lines **(D)**, chemosensitivity **(E)**, and treatment patterns **(F)**.

On the data cutoff day of May 31^st^, 2025, the median PFS and OS for the overall cohort were 10.0 months [95% confidence interval (CI): 8–16 months] and 26.0 months (95% CI: 22–34 months), respectively establishing the real-world efficacy baseline for anlotinib (**Figure 2**). A total of 41 deaths (48.8%) occurred, and 43 patients (51.2%) remained alive, 25 of whom were still receiving anlotinib, including 11 patients in the A+I group. The PFS and OS survival curves according to clinicopathologic characteristics are shown in **Figure 3**; **Supplementary Figure 1**. Subgroup analysis showed that gender, ECOG grade, treatment pattern, HFSR and hypothyroidism were significantly different for both PFS and OS, while body mass was significantly different only for PFS. Additionally, no significant PFS and OS difference was observed among age, FNCLCC grade, tumor size, metastatic sites, treatment lines and chemosensitivity across the entire cohort.

In terms of treatment response, 11 patients (13.1%) showed a partial response (PR), with no complete response (CR), leading to an objective response rate (ORR) of 13.1%. Fifty-eight cases (69.0%) achieved stable disease (SD) and the disease control rate (DCR) was 82.1%. Efficacy varied by treatment pattern. The maintenance therapy group (n=22) achieved the highest DCR (100%) among all subgroups, with SD and PR rates of 81.8% and 18.2% (ORR: 18.2%). The anlotinib plus immunotherapy (A+I) regimen achieved the highest ORR among all patterns (37.5%; 6 PR, 7 SD, 3 PD in 16 patients), with a DCR of 81.2% versus 70.6% for other combinations. Compared with anlotinib monotherapy (median PFS 8 months, OS 20 months; **Table 3**), the A+I group had numerically longer median PFS (20 months) and OS (29 months). The histological distribution across treatment groups is shown in **Supplementary Table 1**. In the A+I group, ASPS and UPS together accounted for 50% (8/16), compared with 20.7%, 11.8%, and 9.1% in the monotherapy, other combination, and maintenance groups, respectively. A descriptive within-group comparison (**Supplementary Figure 2**) showed that these numerically favorable outcomes were predominantly observed in patients with ASPS or UPS. In the A+I group, patients with non-ASPS/UPS histologies had a median PFS of 15 months, although the small subgroup size precludes formal comparison. However, compared with monotherapy, the A+I regimen did not show statistically significant improvement in the adjusted analysis (PFS: HR 0.55, 95% CI 0.25-1.25, *P* = 0.153; OS: HR 0.44, 95% CI 0.15-1.27, *P* = 0.131). The incidence of hypothyroidism was notably higher in the A+I group (81.3%, 13 of 16 patients) compared to the overall cohort (53.6%). According to chemosensitivity profiles (**Table 4**), the hypersensitive subgroup (n=2) achieved a 100% DCR but no objective response. The insensitive subgroup (n=12) showed a numerically higher ORR of 16.7% with a DCR of 75%, and the moderate sensitive subgroup (n=70) had an ORR of 12.9% and a DCR of 82.9%.

**Table 3:** Univariate analysis of survival.

Variable	N	Median PFS (95%CI)	*P* (log-rank)	Median OS (95%CI)	*P* (log-rank)
Age			0.355		0.532
≤40	45	12 (7-20)		24 (20-34)	
>40	39	8 (6-17)		29 (25-NA)	
Gender			0.004^*^		0.006^*^
female	44	16 (13-24)		31 (25-NA)	
male	40	6 (5-10)		22 (13-29)	
ECOG score			0.0168^*^		<0.001^**^
0	36	12 (8-26)		NA	
≥1	48	8 (6-16)		20 (16-29)	
BMI (kg/m^2^)			0.044^*^		0.220
underweight (<18.5)	9	6 (5-NA)		15 (9-NA)	
normal (18.5-24.9)	58	13 (8-20)		27 (24-NA)	
overweight (≥25)	17	8 (6-NA)		25 (14-NA)	
FNCLCC			0.871		0.591
1	5	15 (6-NA)		52 (8-NA)	
2	27	8 (5-21)		29 (20-NA)	
3	52	10 (7-17)		20 (15-31)	
Tumor diameter			0.794		0.753
<5cm	23	13 (8-24)		25 (20-NA)	
5-10cm	39	17 (7-20)		29 (24-NA)	
≥10cm	16	6 (6-NA)		21 (15-NA)	
Pathological type			0.008^*^		0.025^*^
alveolar soft part sarcoma	6	26 (20-NA)		NA	
synovial sarcoma	8	13 (8-NA)		27 (21-NA)	
fibrosarcoma	10	6 (5-NA)		29 (15-NA)	
leiomyosarcoma	14	17 (6-NA)		31 (20-NA)	
UPS	12	5 (4-NA)		16 (9-NA)	
rhabdomyosarcoma	10	7 (4-NA)		17 (11-NA)	
liposarcoma	11	3 (2-NA)		NA	
others	13	15 (10-NA)		25 (22-NA)	
Chemosensitivity			0.209		0.168
insensitive	12	12 (8-19)		29 (23-NA)	
moderate	70	8 (5-17)		26 (16- NA)	
hypersensitive	2	13 (4-NA)		24 (14- NA)	
Metastatic sites			0.220		0.054
intrapulmonary	28	18 (9-26)		29 (27-NA)	
extrapulmonary	27	10 (5-20)		25 (20-NA)	
multiple sites	29	7 (6-17)		20 (15-31)	
Treatment lines of anlotinib			0.654		0.806
1st-line	43	12 (8-19)		29 (23-NA)	
2nd-line	33	8 (5-17)		26 (16- NA)	
≥3rd-line	8	13 (4-NA)		24 (14- NA)	
Treatment pattern of anlotinib			0.002^*^		0.029^*^
monotherapy	29	8 (5-17)		20 (13-NA)	
anlotinib + immunotherapy	16	20 (5-NA)		29 (24-NA)	
other combination therapy	17	5 (3-12)		15 (7-NA)	
maintenance therapy	22	17 (13-NA)		26 (25-NA)	
Surgery history			0.224		0.656
yes	74	10 (8-16)		26 (21-40)	
no	10	12 (6-NA)		27 (16-NA)	
Radiotherapy history			0.711		0.804
yes	37	10 (8-17)		25 (20-NA)	
no	47	11 (6-20)		27 (23-NA)	
Hypothyroidism			<0.001^**^		<0.001^**^
yes	45	17 (13-26)		29 (27-NA)	
no	30	7 (4-13)		21 (13-NA)	
unknown	9	5 (3-NA)		13 (7-NA)	
Hand-foot skin reaction			0.0012^*^		<0.001^**^
yes	51	16 (13-21)		29 (25-NA)	
no	33	6 (4-8)		21 (14-NA)	

NA, not available (median not reached or not estimable); Tumor diameter data were missing in 6 patients (7.1%). ^*^P < 0.05; ^**^P < 0.001.

**Table 4:** Response to anlotinib treatment.

Subgroups	No. of patients (%)		Tumor response				
		CR No. (%)	PR No. (%)	SD No. (%)	PD No. (%)	DCR (%)	ORR (%)
Anlotinib treatment patterns							
monotherapy	29 (34.5%)	0 (0%)	0 (0%)	22 (75.9%)	7 (24.1%)	75.9%	0%
anlotinib+ immunotherapy	16 (19.0%)	0 (0%)	6 (37.5%)	7 (43.8%)	3 (18.8%)	81.2%	37.5%
other combination therapy	17 (20.2%)	0 (0%)	1 (5.9%)	11 (64.7%)	5 (29.4%)	70.6%	5.9%
maintenance therapy	22 (26.2%)	0 (0%)	4 (18.2%)	18 (81.8%)	0 (0%)	100%	18.2%
Metastatic site							
intrapulmonary	28 (33.3%)	0 (0%)	4 (14.3%)	21 (75.0%)	3 (10.7%)	89.3%	14.3%
extrapulmonary	27 (32.1%)	0 (0%)	5 (18.5%)	15 (55.6%)	7 (25.9%)	74.1%	18.5%
multiple sites	29 (34.5%)	0 (0%)	2 (6.9%)	22 (75.9%)	5 (17.2%)	82.8%	6.9%
Chemosensitivity							
insensitive	12 (14.3%)	0 (0%)	2 (16.7%)	7 (58.3%)	3 (25%)	75%	16.7%
moderate	70 (83.3%)	0 (0%)	9 (12.9%)	49 (70%)	12 (17.1%)	82.9%	12.9%
hypersensitive	2 (2.4%)	0 (0%)	0 (0%)	2 (100%)	0 (0%)	100.0%	0%
FNCLCC							
1	5 (6.0%)	0 (0%)	1 (20%)	4 (80%)	0 (0%)	100%	20%
2	27(32.1%)	0 (0%)	1 (3.7%)	22 (81.5%)	4 (14.8%)	85.2%	3.7%
3	52(61.9%)	0 (0%)	9 (17.3%)	32 (61.5%)	11 (21.2%)	78.8%	17.3%
Histological subtype							
alveolar soft part sarcoma	6 (7.1%)	0 (0%)	2 (33.3%)	4 (66.7%)	0 (0%)	100%	33.3%
synovial sarcoma	8 (9.5%)	0 (0%)	0 (0%)	8 (100%)	0 (0%)	100%	0%
fibrosarcoma	10 (11.9%)	0 (0%)	0 (0%)	9 (90%)	1 (10%)	90%	0%
leiomyosarcoma	14 (16.7%)	0 (0%)	1 (7.1%)	11 (78.6%)	2 (14.3%)	85.7%	7.1%
UPS	12 (14.3%)	0 (0%)	4 (33.3%)	4 (33.3%)	4 (33.3%)	66.7%	33.3%
rhabdomyosarcoma	10 (11.9%)	0 (0%)	0 (0%)	8 (80%)	2 (20%)	80%	0%
liposarcoma	11 (13.1%)	0 (0%)	1 (9.1%)	5 (45.5%)	5 (45.5%)	54.5%	9.1%
others	13 (15.5%)	0 (0%)	3 (23.1%)	9 (69.2%)	1 (7.7%)	92.3%	23.1%

CR, complete response; FNCLCC, French Federation of Cancer Centers Sarcoma Group systems; ORR, objective response rate; DCR, disease control rate; PD, progressive disease; PR, partial response; SD, stable disease. undifferentiated pleomorphic sarcoma (UPS)

As shown in **Table 5**, adverse events were common but predominantly low-grade (Grade 1-2). A total of 22 grade ≥3 adverse events were reported (**Table 5**). Among these, six patients required dose reduction and one patient discontinued anlotinib due to a treatment-related pneumothorax. The most frequent adverse events of all grades were hypothyroidism (53.6%), HFSR (60.7%), and proteinuria (51.2%). Sixteen patients temporarily paused treatment until the adverse event resolved; no other treatment-related discontinuations or deaths were observed.

**Table 5:** Adverse events of anlotinib.

Event	Grade 1-2 N (%)	Grade 3-4 N (%)
Hypertension	28 (33.3%)	6 (7.1%)
Diarrhea	15 (17.9%)	0
Proteinuria	41 (48.8%)	2 (2.4%)
Hand-foot skin reaction	48 (57.1%)	3 (3.6%)
Hypothyroidism	45 (53.6%)	0
Cholesterol elevation	21 (25.0%)	2 (2.4%)
Hypertriglyceridemia	31 (36.9%)	2 (2.4%)
ALT elevation	32 (38.1%)	2 (2.4%)
AST elevation	29 (34.5%)	1 (1.2%)
Hyperbilirubinemia	25 (29.8%)	2 (2.4%)
Hyperuricemia	18 (21.4%)	0
Serum creatinine elevation	4 (4.8%)	0
Hypokalemia	6 (7.1%)	0
Thrombocytopenia	2 (2.4%)	0
Arthralgia	19 (22.6%)	0
Hoarseness	8 (9.5%)	0
Abdominal pain	5 (6.0%)	0
Headache	2 (2.4%)	0
Fatigue	23 (27.4%)	0
Mucositis oral	15 (17.9%)	0
Dizziness	4 (4.8%)	0
Nausea	26 (31.0%)	0
Pneumothorax	0	2 (2.4%)

### Prognostic factor analysis

Building upon these efficacy and safety findings, we further investigated prognostic factors associated with PFS and OS to identify clinical predictors of survival outcomes. Factors including gender, age, BMI, ECOG performance score, FNCLCC grade, tumor size, number of metastatic sites, anlotinib treatment patterns, anlotinib treatment lines, chemosensitivity, pathologic type, radiotherapy history, and surgical history at baseline were included in the univariate analysis for PFS and OS. Hypothyroidism and HFSR, the two most common adverse reactions, were also included. The analysis revealed several factors significantly associated with survival outcomes (**Table 3**). Gender, treatment pattern, hypothyroidism and HFSR were significantly correlated with both PFS and OS (all *P* < 0.05). ECOG score (≥1 vs. 0) was also associated with significantly worse PFS (*P* = 0.0168) and OS (*P* < 0.001). Pathological type was a significant factor for both endpoints (PFS: *P* = 0.008; OS: *P* = 0.025). BMI showed a significant association with PFS (*P* = 0.044) but not with OS (*P* = 0.22), primarily driven by poor outcomes in underweight patients (18). The number of metastatic sites exhibited a trend towards association with OS (*P* = 0.054) but not with PFS (*P* = 0.22). In contrast, age, tumor size, FNCLCC grade, chemosensitivity, treatment line, and history of surgery or radiotherapy showed no significant associations (all *P*>0.05).

**Figure 4:**
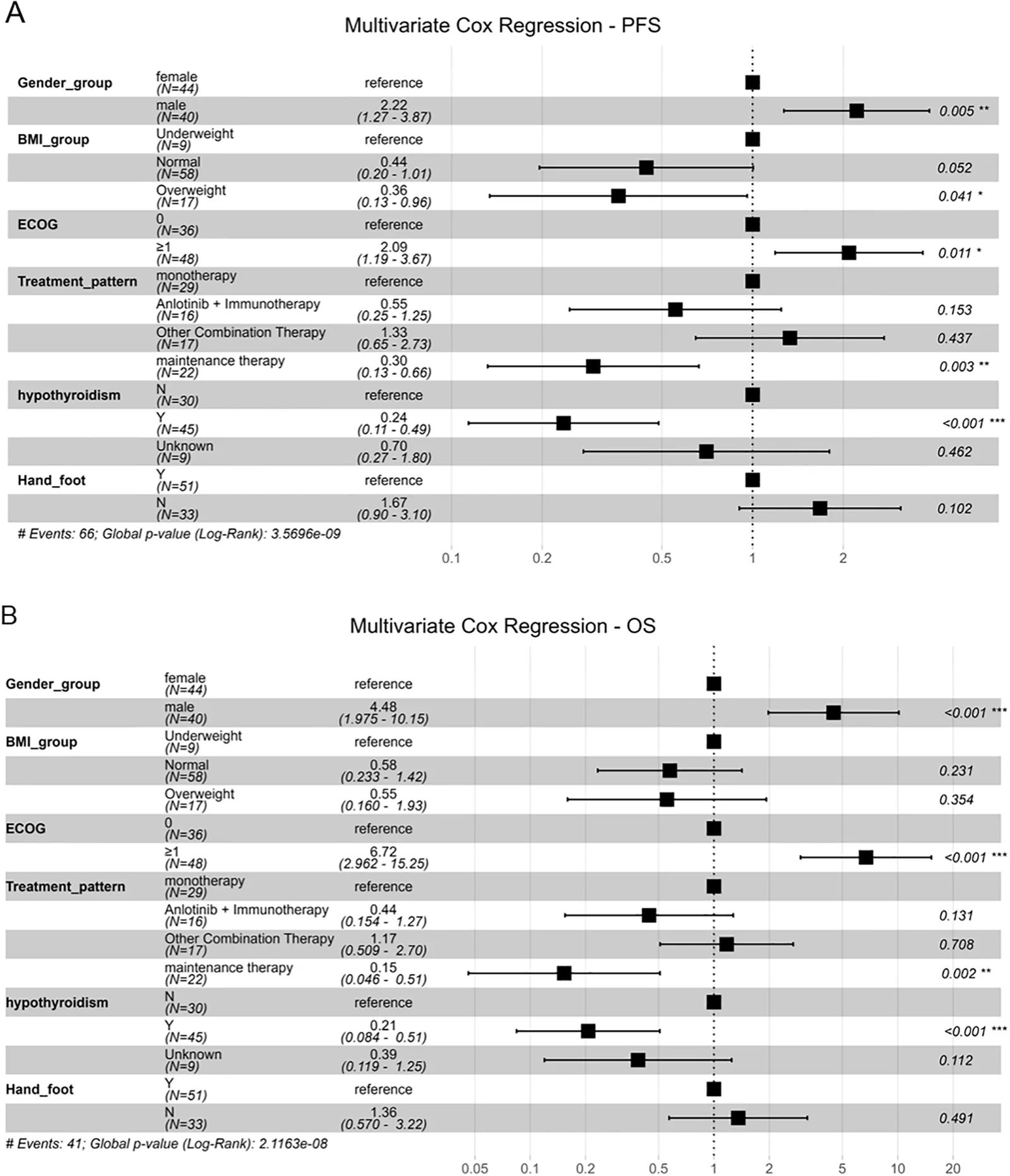
Multivariate Cox regression of progression-free survival **(A)** and overall survival **(B)**.

Based on the above, we incorporated gender, BMI, ECOG score, anlotinib treatment pattern, hypothyroidism and HFSR as variables in multivariable Cox regression analysis, as they represent strong and clinically interpretable prognostic factors across demographic, performance status and therapeutic domains. Pathological subtype was not included due to the multiplicity of categories and limited sample size within individual subtypes, which would compromise the stability and interpretability of the multivariate model. The multivariable analysis confirmed that male gender (PFS HR 2.22, 95% CI 1.27-3.87, *P* = 0.005; OS HR 4.48, 95% CI 1.98-10.15, *P* < 0.001) and ECOG score ≥1 (PFS HR 2.09, 95% CI 1.19-3.67, *P* = 0.011; OS HR 6.72, 95% CI 2.96-15.25, *P* < 0.001) were independent risk factors for worse survival. Conversely, the occurrence of hypothyroidism was a strong independent favorable prognostic factor (PFS HR 0.24, 95% CI 0.11-0.49, *P* < 0.001; OS HR 0.21, 95% CI 0.08-0.51, *P* < 0.001). Notably, HFSR was significant in univariate analysis but did not retain independent prognostic significance in the multivariable model (PFS: *P* = 0.102; OS: *P* = 0.491). This suggests that the prognostic information conveyed by HFSR was largely captured by other covariates, particularly hypothyroidism, possibly reflecting shared biological mechanisms such as treatment intensity rather than an independent association. In an exploratory multivariable Cox model, treatment pattern was associated with differences in PFS (*P* = 0.002) and OS (*P* = 0.029). However, the proportional hazards assumption was not fully satisfied for the treatment pattern variable. In this model, maintenance therapy showed an association with longer PFS (HR 0.30, 95% CI 0.13-0.66, *P* = 0.003) and OS (HR 0.15, 95% CI 0.05-0.51, *P* = 0.002) compared with monotherapy, but these estimates should be interpreted as exploratory rather than confirmatory because they could not be independently validated in the stratified Cox model. It also showed a notably long median PFS (17 months) and median OS (26 months). Anlotinib plus immunotherapy demonstrated the most favorable descriptive outcomes in survival estimates among all treatment patterns, but the adjusted comparison against monotherapy was not statistically significant for either endpoint. This suggests that the numerically longer survival may be influenced by histological composition and other prognostic factors, or limited by sample size. Other combination therapy (anlotinib with chemotherapy) was associated with the shortest median PFS (5 months) and median OS (15 months) among all treatment patterns. Notably, these outcomes remain comparable to historical data for anlotinib monotherapy (median PFS 6.27 months in the ALTER0203 trial (8)), likely reflecting the more heavily pretreated nature of patients receiving later-line chemotherapy combinations. These results collectively suggest that treatment pattern may be associated with survival differences in exploratory analyses, but these findings require cautious interpretation. The complete results of the multivariable analysis are presented in **Figure 4**; **Supplementary Table 2**.

The PFS model was based on 66 events and the OS model on 41 events (**Figure 4**). All VIFs were below 2.5. The Schoenfeld test indicated some departure from the proportional hazards assumption in the primary model (PFS global *P* = 0.008; OS global *P* < 0.001), primarily attributable to the treatment pattern variable. After fitting a stratified Cox model with treatment pattern as the stratification variable, the proportional hazards assumption was satisfied (PFS global *P* = 0.34; OS global *P* = 0.73), and the results were largely consistent with the primary analysis for the key prognostic factors, except for BMI in the OS model (**Table 6**). In a sensitivity analysis additionally adjusting for histological grouping (ASPS/UPS vs. other histologies), the hazard ratios for the A+I regimen were attenuated (PFS: HR 0.63, 95% CI 0.26-1.50; OS: HR 0.56, 95% CI 0.18-1.75; **Supplementary Table 3**), whereas the estimates for maintenance therapy and hypothyroidism remained stable. Ridge regression coefficients were in the same direction as the primary Cox model (e.g., PFS: A+I -0.41, maintenance -0.65, hypothyroidism-N 0.84; **Supplementary Table 4**), supporting the robustness of the findings. Baseline characteristics by hypothyroidism status are shown in **Supplementary Table 5**. Age, sex, ECOG performance status, metastatic sites, and treatment line were relatively balanced (SMD < 0.3), whereas BMI, histological subtype, and treatment pattern showed notable imbalances (SMD > 0.5), FNCLCC grade showed mild imbalance (SMD 0.335).

**Table 6:** Stratified Cox model with treatment pattern as the stratification variable, compared with the primary multivariable model.

Variable	Primary PFS HR (95% CI)	*P*	Stratified PFS HR (95% CI)	*P*	Primary OS HR (95% CI)	*P*	Stratified OS HR (95% CI)	*P*
Male vs. Female	2.22 (1.27–3.87)	0.005	2.24 (1.28–3.93)	0.005	4.48 (1.98–10.15)	<0.001	4.21 (1.81–9.80)	<0.001
BMI Normal vs. Underweight	0.44 (0.20–1.01)	0.052	0.47 (0.20–1.11)	0.086	0.58 (0.23–1.42)	0.231	0.32 (0.12–0.85)	0.021
BMI Overweight vs. Underweight	0.36 (0.13–0.99)	0.041	0.40 (0.14–1.10)	0.076	0.55 (0.16–1.93)	0.355	0.28 (0.08–1.01)	0.052
ECOG ≥1 vs. 0	2.09 (1.19–3.67)	0.011	2.08 (1.14–3.78)	0.017	6.72 (2.96–15.25)	<0.001	6.67 (2.77–16.03)	<0.001
A+I vs. Monotherapy	0.55 (0.25–1.25)	0.153			0.44 (0.15–1.27)	0.131		
Other combination vs. Monotherapy	1.33 (0.65–2.73)	0.437			1.17 (0.51–2.70)	0.708		
Maintenance vs. Monotherapy	0.30 (0.13–0.66)	0.003			0.15 (0.05–0.51)	0.002		
Hypothyroidism N vs. Y	4.24 (2.05–8.79)	<0.001	3.17 (1.57–6.37)	0.001	4.84 (1.97–11.86)	<0.001	4.94 (1.87–13.06)	0.001
Hypothyroidism Unknown vs. Y	2.98 (1.07–8.29)	0.036	2.53 (0.91–7.00)	0.075	1.87 (0.55–6.36)	0.318	1.85 (0.52–6.59)	0.341
HFSR N vs. Y	1.67 (0.90–3.10)	0.102	1.61 (0.85–3.03)	0.141	1.36 (0.57–3.22)	0.491	1.09 (0.45–2.65)	0.844

The stratified model does not produce hazard ratios for the stratification variable itself (treatment pattern). The Schoenfeld test was satisfied in the stratified model (PFS global *P* = 0.34; OS global *P* = 0.73). Primary multivariable Cox model (PFS global *P* = 0.008; OS global *P* < 0.001).

### Sensitivity analysis

The robustness of hypothyroidism as a prognostic factor was evaluated by landmark analyses at 2, 3, and 4 months. As shown in **Table 1**, after excluding patients with PFS<2 months (n = 83, 98.8% of the original cohort), hypothyroidism remained strongly associated with prolonged PFS (HR 0.21, 95% CI 0.10-0.45, *P* < 0.001) and OS (HR 0.19, 95% CI 0.08-0.47, *P* < 0.001). Similar results were observed at 3 months (n=72): PFS HR 0.25 (95% CI 0.11-0.57, *P* = 0.001) and OS HR 0.32 (95% CI 0.11-0.94, *P* = 0.038); and at 4 months (n=67): PFS HR 0.22 (95% CI 0.09-0.50, *P* < 0.001) and OS HR 0.30 (95% CI 0.10-0.91, *P* = 0.033). These consistent findings across multiple time points confirm that the prognostic value of hypothyroidism is not driven by immortal time bias. Excluding patients with unknown hypothyroidism status (n=9) from the primary multivariable Cox model (without additional adjustment for histological grouping) did not materially change the results; compared with patients who developed hypothyroidism, those without hypothyroidism had significantly worse survival (PFS: HR 4.20, 95% CI 1.98-8.91, *P* = 0.0002; OS: HR 4.62, 95% CI 1.82-11.75, *P* = 0.0013).

## Discussion

For patients with advanced soft tissue sarcoma, optimizing treatment strategies remains a critical clinical challenge. This real-world study of 84 patients treated with anlotinib-based regimens identified three findings: the A+I combination showed numerically the longest survival among treatment patterns in a subgroup with a higher proportion of immunotherapy-sensitive subtypes, maintenance therapy was associated with prolonged survival, and treatment-emergent hypothyroidism emerged as a novel independent favorable prognostic factor. These observations may inform future research on anti-angiogenic therapy in advanced STS.

Distinct treatment patterns were associated with varying degrees of clinical benefit. The A+I combination showed numerically the longest survival, with a median PFS of 20 months, a median OS of 29 months, and an ORR of 37.5%, substantially exceeding both the historical ORR of approximately 10% for anlotinib monotherapy in the ALTER0203 trial (8) and the overall population ORR of 13.1% in our cohort. Notably, the 16 patients who received A+I in our cohort predominantly presented with ASPS and UPS-subtypes previously reported to be more responsive to both immunotherapy and anti-angiogenic therapy (11, 15). This patient composition likely contributed substantially to the favorable outcomes observed with the A+I regimen, and the results should therefore be interpreted with caution. Nevertheless, we also observed disease control in individual cases of other subtypes, including liposarcoma, synovial sarcoma, and fibrosarcoma, suggesting that the A+I combination may have activity beyond ASPS/UPS. These descriptive findings suggest that the addition of PD-1 inhibitors to anlotinib may warrant further investigation in selected STS patients, particularly those with immunologically active subtypes, though prospective validation in larger cohorts is needed.

Anlotinib maintenance therapy was associated with prolonged survival, but this finding should be interpreted with substantial caution. Patients in this group were selected for having achieved disease control after chemotherapy, which confers a favorable prognosis. Moreover, the proportional hazards assumption was not fully satisfied for the treatment pattern variable, and the stratified Cox model could not provide hazard ratios for treatment pattern, preventing independent validation of these estimates. Therefore, the observed association may reflect selection bias and model limitations rather than a causal effect of maintenance anlotinib. These results support the feasibility of this approach in appropriately selected patients but do not demonstrate superiority over other strategies. The median PFS of 17 months in our maintenance group compares favorably with the 9.1 months reported in the prospective phase II ALTER-S006 trial (10). While this difference may partly reflect the inherent heterogeneity of real-world populations, it also suggests that for patients achieving disease control after first-line chemotherapy, subsequent anlotinib maintenance may achieve not only delayed progression but prolonged disease control in routine clinical practice compared to controlled trial settings. Notably, although the A+I regimen demonstrated numerically longer median PFS and OS than maintenance therapy, the A+I regimen did not show a statistically significant improvement over monotherapy in adjusted analysis, and its favorable outcomes were likely influenced by the higher proportion of immunologically active subtypes such as ASPS and UPS. For anlotinib monotherapy, the median PFS of 8 months compared favorably with the 6.27 months in the ALTER0203 trial (8) and historical first-line chemotherapy data (4–6), reinforcing its role as a valuable non-chemotherapy option for patients ineligible for or intolerant to cytotoxic agents.

Beyond treatment patterns, we also examined patient-intrinsic prognostic factors. Male gender and poor ECOG performance status were independent risk factors for worse survival. The prognostic value of ECOG performance status has been consistently reported in real-world STS studies (19), reinforcing its utility as a stratification factor. The observed sex-based survival difference is consistent with recent evidence of sex-dependent prognosis in advanced STS, particularly in the context of immunotherapy (20–22), and may reflect estrogen-mediated regulation of the VEGF pathway (23) and sex differences in immune responses (24). The lack of a significant association between FNCLCC grade and survival may reflect several factors, including the histological heterogeneity of the cohort, the limited sample size (n=84) with an unbalanced grade distribution (grade 1: n=5; grade 2: n=27; grade 3: n=52), and the retrospective single-center design. These limitations may have reduced statistical power to detect an association, and therefore this negative finding should be interpreted with caution. The baseline characteristics of our patients were consistent with previously reported real-world STS cohorts (19), supporting the external validity of our observations. The safety profile aligned with the known characteristics of anlotinib, with no new safety signals identified (25), confirming its favorable tolerability. The most common adverse events were HFSR, hypothyroidism, and proteinuria, predominantly low-grade. The low dose reduction rate indicates that, with meticulous management, anlotinib is suitable for long-term administration. Treatment-emergent hypothyroidism was independently associated with favorable survival, and its incidence was notably higher in the A+I group than in the overall cohort. It is important to distinguish between a prognostic and a predictive marker: our data identify hypothyroidism as a favorable prognostic factor in patients receiving anlotinib-based therapy, but cannot determine whether it specifically predicts greater benefit from PD-1 inhibitor addition. While hypothyroidism is a recognized on-target effect of VEGFR inhibition (7), the mechanisms underlying the association with prolonged survival remain unclear. Hypothyroidism may result from anlotinib alone, PD-1 inhibition, pre-existing thyroid conditions, treatment duration, or differences in monitoring frequency. These possible explanations should be regarded as hypotheses rather than established mechanisms, and the retrospective nature of the data precludes causal inference. Indeed, thyroid dysfunction during anti-PD-1 therapy has been reported to predict improved survival (26). In STS, where anti-PD-1 responses are subtype-dependent (11), whether treatment-emergent hypothyroidism can serve as a predictive marker requires further investigation. This association persisted across multiple landmark analyses, mitigating the potential impact of immortal time bias. The association also remained stable when patients with unknown thyroid status were excluded from the analysis. Comparison of baseline characteristics by hypothyroidism status showed that the two groups were broadly similar in established prognostic factors, including age, sex, and ECOG performance status.

The robustness of the multivariable findings was further evaluated. Adjustment for histological grouping attenuated the association between the A+I regimen and survival, reinforcing the interpretation that the favorable outcomes in this subgroup were largely driven by histology. In contrast, the associations of maintenance therapy and hypothyroidism with survival remained stable across these analyses. Some departure from the proportional hazards assumption was noted in the primary model, mainly related to the treatment pattern variable. To address this, we fitted a stratified Cox model with treatment pattern as the stratification variable, which relaxes the proportional hazards assumption across treatment groups. Although this stratified model satisfied the proportional hazards assumption for the remaining covariates, it could not provide hazard ratios for treatment pattern itself, and therefore could not independently validate the treatment-specific estimates from the primary model. Given the limited number of events, time-varying effect analysis was not feasible. Consequently, all treatment pattern hazard ratios should be considered exploratory and not evidence of a causal survival benefit. Ridge penalized regression, performed as an additional sensitivity check for coefficient stability, yielded estimates consistent in direction with the primary Cox model. Taken together, these analyses support the robustness of our findings, particularly the independent prognostic value of treatment-emergent hypothyroidism.

This study has several limitations. First, the retrospective, single-center design may introduce selection bias; baseline imbalances across treatment groups were observed, particularly in histological subtype, which should be considered when interpreting the treatment comparisons. Furthermore, the apparent benefit of maintenance therapy is confounded by indication: patients who achieve disease control on prior chemotherapy have more favorable disease biology, and the observed survival benefit should not be interpreted as a causal effect of maintenance anlotinib. Detailed data on the number of prior chemotherapy cycles, best response before anlotinib, and the exact interval from chemotherapy initiation to anlotinib start were not individually recorded; however, all maintenance patients had achieved at least stable disease by definition, which partially reflects treatment sensitivity. Given the low incidence of STS, these findings require validation in larger multicenter prospective cohorts. Second, the A+I regimen demonstrated the highest ORR and numerically the longest survival outcomes among all treatment patterns, though the adjusted comparisons did not reach statistical significance in multivariable analysis. This may be attributable to the small size of the A+I subgroup and potential confounding by other prognostic factors, such as the higher proportion of immunologically active subtypes (ASPS and UPS). Nevertheless, the observation that non-ASPS/UPS patients in the A+I group also achieved a median PFS of 15 months, while limited by the very small subgroup size and lack of a matched control, raises the possibility that histology may not be the sole driver of the observed benefit. This warrants further evaluation in biomarker-driven or histology-stratified prospective trials. Third, the median age of 30 years reflects the inclusion of young-adult subtypes such as ASPS and rhabdomyosarcoma. Three patients under 18 years of age (aged 11, 16, and 17 years) were included. All were managed under adult sarcoma protocols with standard adult dosing. Given the small number of minor patients, a formal assessment of their specific impact on treatment selection and outcomes is not feasible, and the possibility that these cases influenced the observed age distribution and survival estimates cannot be excluded. Although this histological heterogeneity and young age distribution may limit direct extrapolation to older, unselected adult STS populations, our findings provide real-world evidence for anlotinib-based regimens in a younger, histologically diverse cohort and highlight clinically relevant prognostic factors. The results should therefore be interpreted with caution when applied to advanced STS as a single disease entity, but they generate hypotheses for future prospective studies in more homogeneous subgroups. In addition, the number of patients in the hypersensitive group was small (n=2), which reflects the low incidence of highly chemosensitive subtypes in the adult STS population, rather than selection bias. This grouping was intended to describe expected chemosensitivity based on histology rather than to support statistical comparisons, and results for this subgroup should be interpreted with caution. The date of hypothyroidism onset was not systematically recorded, precluding time-dependent analysis; therefore, the temporal relationship between hypothyroidism and survival could not be further characterized. In addition, pre-existing thyroid conditions and differences in the frequency of thyroid function monitoring across treatment groups could not be fully accounted for, which may have influenced the detection of hypothyroidism. Data on subsequent therapies after anlotinib progression were not systematically recorded, and their potential influence on OS could not be assessed. Due to the relatively limited sample size and the influence of other prognostic factors, further verification is needed.

## Conclusions

In this real-world study, the addition of PD-1 inhibitors to anlotinib was associated with numerically longer survival and a high disease control rate in a small subgroup with a higher proportion of immunotherapy-sensitive histologies; these descriptive findings warrant further prospective evaluation. Treatment-emergent hypothyroidism was identified as an independent favorable prognostic factor. Maintenance therapy was associated with prolonged survival, but this finding should be interpreted cautiously due to potential selection bias and violation of the proportional hazards assumption. The prognostic value of hypothyroidism was consistent across multiple landmark analyses, mitigating the potential impact of immortal time bias. These observations provide a rationale for prospective validation in larger, histologically balanced cohorts.

## Data Availability

The raw data supporting the conclusions of this article will be made available by the authors, without undue reservation.

## Supplementary material

Supplementary Figure 1 Survival curves of progression-free survival and overall survival according to age **(A)**, FNCLCC grade **(B)**, tumor size **(C)**, metastatic sites**(D)**, hand-foot**(E)**, and hypothyroidism **(F)**.

Supplementary Figure 2 Descriptive Kaplan-Meier curves of PFS and OS in the A+I group, stratified by histology (ASPS/UPS, n=8 vs. other histologies, n=8). Because of the very small subgroup sizes, no P values or confidence intervals are shown.
