# Supplementary material for: Anlotinib plus PD-1 inhibitor in advanced soft tissue sarcoma: a real-world study on treatment patterns and treatment-emergent hypothyroidism
Source: Front Immunol. 2026 Sep 11;17:1949174. doi: 10.3389/fimmu.2026.1949174 (PMC13612474; doi:10.3389/fimmu.2026.1949174)
Supplement: Supplementary file 1 [file Table1.docx]

**Supplementary Table S1 Baseline characteristics stratified by treatment pattern**

| **Characteristic** | **Monotherapy (n=29)** | **A+I**  **(n=16)** | **Other combination (n=17)** | **Maintenance (n=22)** | **SMD** |
| --- | --- | --- | --- | --- | --- |
| **Age (%)** |  |  |  |  | 0.232 |
| ≤40 | 17 (58.6) | 6 (37.5) | 10 (58.8) | 12 (54.5) |  |
| >40 | 12 (41.4) | 10 (62.5) | 7 (41.2) | 10 (45.5) |  |
| **Gender (%)** |  |  |  |  | 0.148 |
| female | 14 (48.3) | 9 (56.2) | 8 (47.1) | 13 (59.1) |  |
| male | 15 (51.7) | 7 (43.8) | 9 (52.9) | 9 (40.9) |  |
| **ECOG (%)** |  |  |  |  | 0.070 |
| 0 | 12 (41.4) | 7 (43.8) | 8 (47.1) | 9 (40.9) |  |
| ≥1 | 17 (58.6) | 9 (56.2) | 9 (52.9) | 13 (59.1) |  |
| **BMI (%)** |  |  |  |  | 0.349 |
| underweight | 4 (13.8) | 1 (6.2) | 3 (17.6) | 1 (4.5) |  |
| normal | 18 (62.1) | 13 (81.2) | 10 (58.8) | 17 (77.3) |  |
| overweight | 7 (24.1) | 2 (12.5) | 4 (23.5) | 4 (18.2) |  |
| **Histological type (%)** |  |  |  |  | 1.117 |
| ASPS | 1 (3.4) | 4 (25.0) | 1 (5.9) | 0 (0.0) |  |
| synovial sarcoma | 3 (10.3) | 1 (6.2) | 2 (11.8) | 2 (9.1) |  |
| fibrosarcoma | 4 (13.8) | 1 (6.2) | 3 (17.6) | 2 (9.1) |  |
| leiomyosarcoma | 8 (27.6) | 1 (6.2) | 1 (5.9) | 4 (18.2) |  |
| UPS | 5 (17.2) | 4 (25.0) | 1 (5.9) | 2 (9.1) |  |
| rhabdomyosarcoma | 2 (6.9) | 0 (0.0) | 3 (17.6) | 5 (22.7) |  |
| liposarcoma | 3 (10.3) | 2 (12.5) | 5 (29.4) | 1 (4.5) |  |
| others | 3 (10.3) | 3 (18.8) | 1 (5.9) | 6 (27.3) |  |
| **FNCLCC (%)** |  |  |  |  | 0.461 |
| 1 | 0 (0.0) | 1 (6.2) | 1 (5.9) | 3 (13.6) |  |
| 2 | 13 (44.8) | 3 (18.8) | 6 (35.3) | 5 (22.7) |  |
| 3 | 16 (55.2) | 12 (75.0) | 10 (58.8) | 14 (63.6) |  |
| **Metastatic sites (%)** |  |  |  |  | 0.419 |
| intrapulmonary | 10 (34.5) | 5 (31.2) | 3 (17.6) | 10 (45.5) |  |
| extrapulmonary | 8 (27.6) | 4 (25.0) | 8 (47.1) | 7 (31.8) |  |
| multiple Sites | 11 (37.9) | 7 (43.8) | 6 (35.3) | 5 (22.7) |  |
| **Treatment lines** |  |  |  |  | 0.566 |
| 1st-line | 13 (44.8) | 5 (31.2) | 8 (47.1) | 17 (77.3) |  |
| 2nd-line | 13 (44.8) | 8 (50.0) | 7 (41.2) | 5 (22.7) |  |
| ≥3rd-line | 3 (10.3) | 3 (18.8) | 2 (11.8) | 0 (0.0) |  |
| **Surgery history (%)** |  |  |  |  | 0.338 |
| Y | 28 (96.6) | 12 (75.0) | 15 (88.2) | 19 (86.4) |  |
| N | 1 (3.4) | 4 (25.0) | 2 (11.8) | 3 (13.6) |  |
| **Radiotherapy history (%)** |  |  |  |  | 0.201 |
| Y | 13 (44.8) | 5 (31.2) | 8 (47.1) | 11 (50.0) |  |
| N | 16 (55.2) | 11 (68.8) | 9 (52.9) | 11 (50.0) |  |

**SMD: standardized mean difference.

**Supplementary Table S2 Multivariable Cox regression analysis for PFS and OS (corresponding to Figure 4)**

| **Variable** | **PFS HR (95% CI)** | **PFS P value** | **OS HR (95% CI)** | **OS P value** |
| --- | --- | --- | --- | --- |
| **Gender** |  |  |  |  |
| female | 1.00 (ref) |  | 1.00 (ref) |  |
| male | 2.22 (1.27-3.87) | 0.005 | 4.48 (1.98‑10.15) | <0.001 |
| **BMI** |  |  |  |  |
| underweight | 1.00 (ref) |  | 1.00 (ref) |  |
| normal | 0.44 (0.20‑1.01) | 0.052 | 0.58 (0.23‑1.42) | 0.231 |
| overweight | 0.36 (0.13-0.99) | 0.041 | 0.55 (0.16‑1.93) | 0.355 |
| **ECOG** |  |  |  |  |
| 0 | 1.00 (ref) |  | 1.00 (ref) |  |
| ≥1 | 2.09 (1.19-3.67) | 0.011 | 6.72 (2.96‑15.25) | <0.001 |
| **Treatment pattern** |  |  |  |  |
| monotherapy | 1.00 (ref) |  | 1.00 (ref) |  |
| A+I | 0.55 (0.25-1.25) | 0.153 | 0.44 (0.15‑1.27) | 0.131 |
| other combination | 1.33 (0.65‑2.73) | 0.437 | 1.17 (0.51‑2.70) | 0.708 |
| maintenance | 0.30 (0.13-0.66) | 0.003 | 0.15 (0.05‑0.51) | 0.002 |
| **Hypothyroidism** |  |  |  |  |
| N | 1.00 (ref) |  | 1.00 (ref) |  |
| Y | 0.24 (0.11‑0.49) | <0.001 | 0.21 (0.084‑0.51) | <0.001 |
| unknown | 0.70 (0.27‑1.80) | 0.462 | 0.39 (0.119‑1.25) | 0.112 |
| **HFSR** |  |  |  |  |
| Y | 1.00 (ref) |  | 1.00 (ref) |  |
| N | 1.67 (0.90‑3.10) | 0.102 | 1.36 (0.57‑3.22) | 0.491 |

HR: hazard ratio; CI: confidence interval; PFS: progression‑free survival; OS: overall survival.

**Supplementary Table S3 Sensitivity analysis-multivariable model with additional adjustment for histological group**

| **Variable** | **PFS HR (95% CI)** | **OS HR (95% CI)** |
| --- | --- | --- |
| Gender (male vs. female) | 2.25 (1.29‑3.92) | 4.90 (2.09‑11.47) |
| BMI: Normal vs. Underweight | 0.42 (0.18‑0.95) | 0.51 (0.20‑1.30) |
| BMI: Overweight vs. Underweight | 0.34 (0.13‑0.92) | 0.55 (0.16‑1.89) |
| ECOG (≥1 vs. 0) | 2.08 (1.18‑3.64) | 6.72 (2.97‑15.17) |
| A+I vs. Monotherapy | 0.63 (0.26‑1.50) | 0.56 (0.18‑1.75) |
| Other combination vs. Monotherapy | 1.30 (0.63‑2.68) | 1.20 (0.52‑2.79) |
| Maintenance vs. Monotherapy | 0.29 (0.13‑0.65) | 0.15 (0.04‑0.48) |
| Hypothyroidism: N vs. Y | 4.31 (2.08‑8.94) | 4.91 (1.99‑12.09) |
| Hypothyroidism: Unknown vs. Y | 3.05 (1.10‑8.47) | 1.93 (0.58‑6.44) |
| HFSR (N vs. Y) | 1.66 (0.90‑3.07) | 1.34 (0.57‑3.12) |
| Histology (ASPS/UPS vs. Other) | 0.78 (0.39‑1.56) | 0.65 (0.25‑1.67) |

**Supplementary Table S4 Cox model vs. Ridge regression coefficients**

| Variable | Cox PFS coef | Ridge PFS coef | Cox OS coef | Ridge OS coef |
| --- | --- | --- | --- | --- |
| Male vs. Female | 0.80 | 0.56 | 1.50 | 0.92 |
| BMI Normal vs. Under | –0.81 | –0.37 | –0.55 | –0.34 |
| BMI Overweight vs. Under | –1.03 | –0.45 | –0.59 | –0.32 |
| ECOG ≥1 vs. 0 | 0.74 | 0.49 | 1.91 | 1.22 |
| A+I vs. Monotherapy | –0.59 | –0.41 | –0.81 | –0.51 |
| Other combination vs. Monotherapy | 0.29 | 0.44 | 0.16 | 0.36 |
| Maintenance vs. Monotherapy | –1.22 | –0.65 | –1.88 | –0.91 |
| Hypothyroidism N vs. Y | 1.45 | 0.84 | 1.58 | 0.93 |
| Hypothyroidism Unknown vs. Y | 1.09 | 0.69 | 0.62 | 0.50 |
| HFSR N vs. Y | 0.52 | 0.50 | 0.30 | 0.44 |

**Supplementary Table S5 Baseline characteristics by hypothyroidism status**

| **Characteristic** | **Hypothyroidism (n=45)** | **No hypothyroidism (n=30)** | **SMD** |
| --- | --- | --- | --- |
| **Age (%)** |  |  | <0.001 |
| ≤40 | 24 (53.3) | 16 (53.3) |  |
| >40 | 21 (46.7) | 14 (46.7) |  |
| **Gender (%)** |  |  | 0.135 |
| female | 27 (60.0) | 16 (53.3) |  |
| male | 18 (40.0) | 14 (46.7) |  |
| **ECOG (%)** |  |  | 0.180 |
| 0 | 22 (48.9) | 12 (40.0) |  |
| ≥1 | 23 (51.1) | 18 (60.0) |  |
| **BMI (%)** |  |  | 0.554 |
| underweight | 6 (13.3) | 3 (10.0) |  |
| normal | 35 (77.8) | 18 (60.0) |  |
| overweight | 4 (8.9) | 9 (30.0) |  |
| **Histological type (%)** |  |  | 0.875 |
| ASPS | 6 (13.3) | 0 (0.0) |  |
| synovial sarcoma | 4 (8.9) | 3 (10.0) |  |
| fibrosarcoma | 5 (11.1) | 4 (13.3) |  |
| leiomyosarcoma | 10 (22.2) | 2 (6.7) |  |
| UPS | 5 (11.1) | 6 (20.0) |  |
| rhabdomyosarcoma | 2 (4.4) | 5 (16.7) |  |
| liposarcoma | 6 (13.3) | 4 (13.3) |  |
| others | 7 (15.6) | 6 (20.0) |  |
| **FNCLCC (%)** |  |  | 0.335 |
| 1 | 4 (8.9) | 1 (3.3) |  |
| 2 | 16 (35.6) | 8 (26.7) |  |
| 3 | 25 (55.6) | 21 (70.0) |  |
| **Metastatic sites (%)** |  |  | 0.241 |
| intrapulmonary | 15 (33.3) | 9 (30.0) |  |
| extrapulmonary | 13 (28.9) | 12 (40.0) |  |
| multiple Sites | 17 (37.8) | 9 (30.0) |  |
| **Treatment lines** |  |  | 0.213 |
| 1st-line | 23 (51.1) | 14 (46.7) |  |
| 2nd-line | 17 (37.8) | 14 (46.7) |  |
| ≥3rd-line | 5 (11.1) | 2 (6.7) |  |
| **Treatment pattern** |  |  | 0.676 |
| monotherapy | 16 (35.6) | 8 (26.7) |  |
| anlotinib + immunotherapy | 13 (28.9) | 3 (10.0) |  |
| other combination therapy | 5 (11.1) | 9 (30.0) |  |
| maintenance therapy | 11 (24.4) | 10 (33.3) |  |

SMD, standardized mean difference. Patients with unknown hypothyroidism status (n=9) were excluded from this comparison.
